# Supplementary material for: Artificial Light at Night Drives Earlier Singing in a Neotropical Bird
Source: Animals (Basel). 2022 Apr 13;12(8):1015. doi: 10.3390/ani12081015 (PMC9028044; doi:10.3390/ani12081015)
Supplement: Supplementary file 1 [file animals-12-01015-s001.zip › animals-1656621-supplementary.pdf]

animals

MDPI

Supplementary materials

Artificial Light at Night Drives Earlier Singing in a Neotropical Bird

Oscar Humberto Marín Gómez [1,2]

[1] Programa de Biología. Grupo de Biodiversidad y Educación Ambiental, Grupo de Investigación y Asesoría en Estadística, Carrera 15 #12N, Armenia, 630004, Colombia; oschumar@iztacala.unam.mx

[2] Laboratorio de Ecología, Unidad de Biotecnología y Prototipos (UBIPRO), Facultad de Estudios Superiores Iztacala Universidad Nacional Autónoma de México, Av. de los Barrios 1, Los Reyes Iztacala, Tlalnepantla 54090, Mexico

Citation: Marín Gómez, O.H. Artificial Light at Night Drives Earlier Singing in a Neotropical Bird. *Animals* **2022**, *11*, 1015. https://doi.org/10.3390/ani12081015

Academic Editor: Kylie Robert

Received: 13 March 2022
Accepted: 12 April 2022
Published: 13 April 2022

Publisher's Note: MDPI stays neutral with regard to jurisdictional claims in published maps and institutional affiliations.

Copyright: © 2022 by the authors. Submitted for possible open access publication under the terms and conditions of the Creative Commons Attribution (CC BY) license (https://creativecommons.org/licenses/by/4.0/).

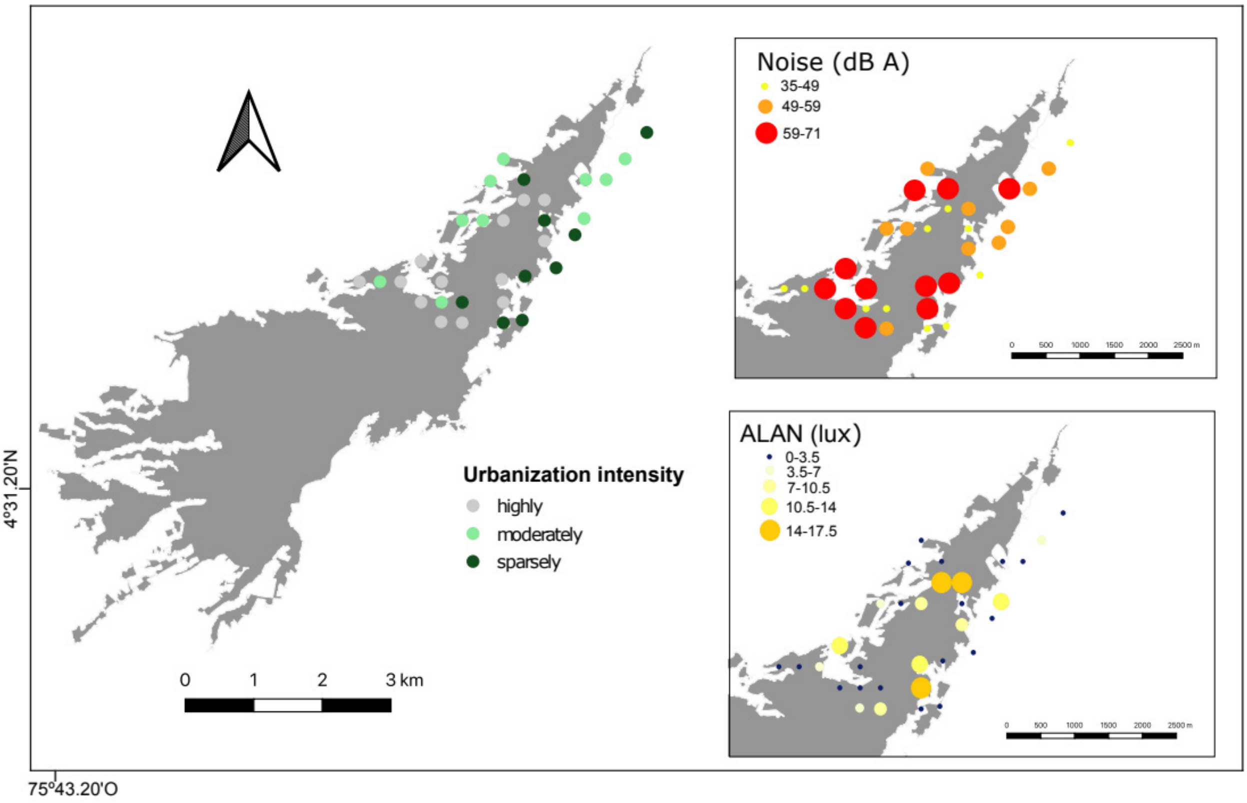

Figure S1. Map of the Armenia city showing the studied locations according to urbanization intensity, anthropogenic noise levels a and artificial light levels at dawn.

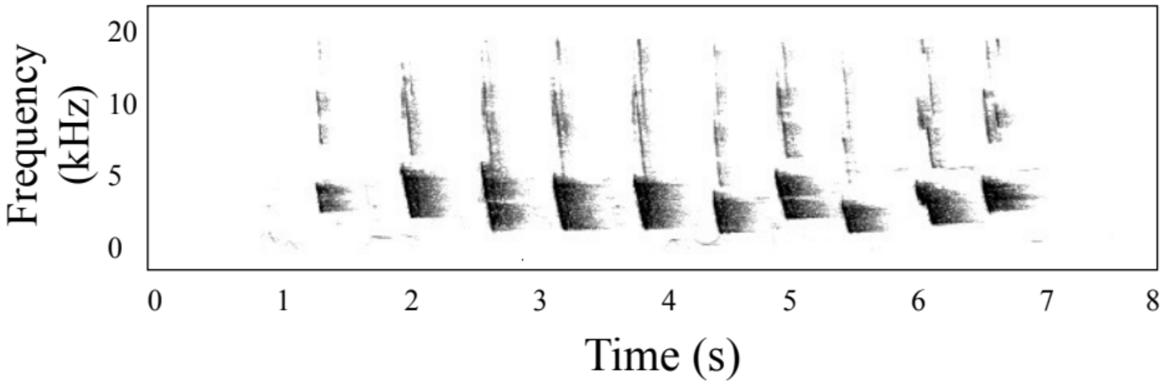

Figure S2. Spectrogram of the dawn song of the Saffron Finch recorded in this study.

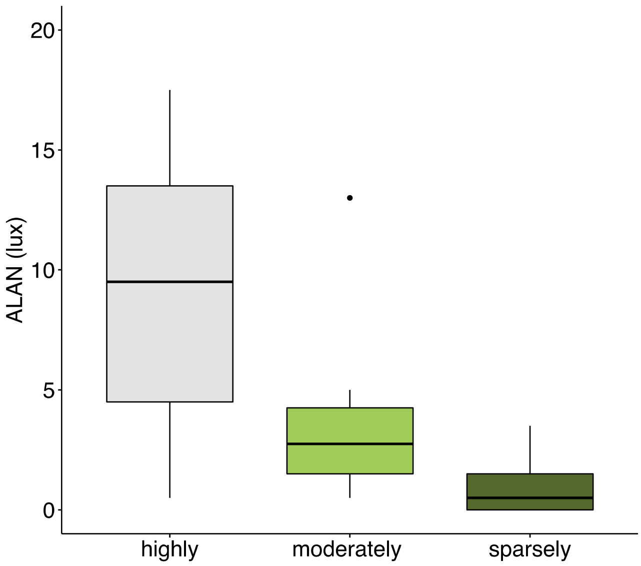

Figure S3. Variation of ALAN levels among urbanization conditions in the Armenia city, Colombia. The outlier point in the moderately urbanization condition represents a sampling point just under the light poles.

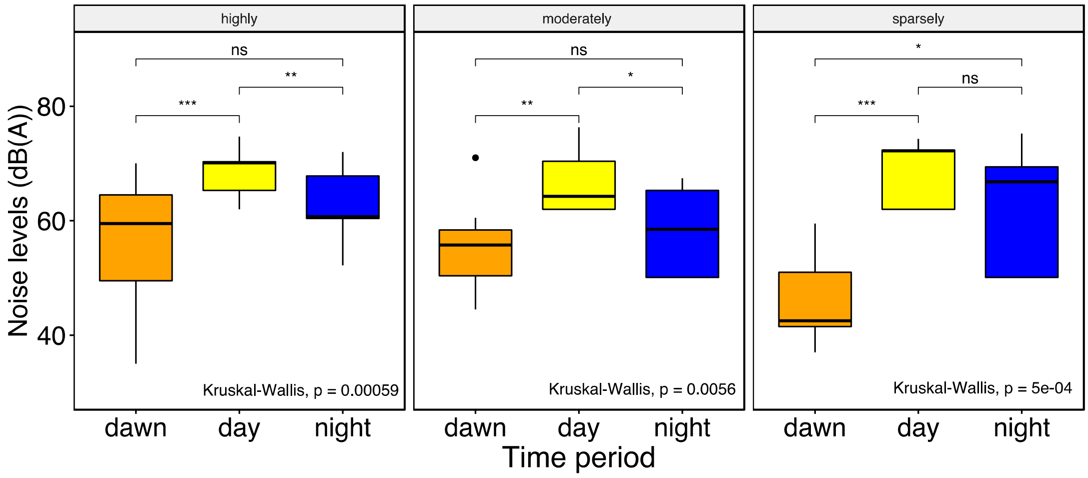

Figure S4. Variation of anthropogenic noise levels among urbanization conditions in the Armenia city, Colombia. The outlier point in the moderately urbanization condition represents a sampling point under high levels of traffic. * $p < 0.05$, ** $p < 0.01$, *** $p < 0.001$.
